# Chinese herbal medicine Yanghe decoction for ankylosing spondylitis: A protocol for systematic review and meta-analysis

**DOI:** 10.1097/MD.0000000000023466

**Published:** 2020-11-25

**Authors:** Xiaosheng Xu, Hong Chen, Xiaoping Yuan, Yi Wan, Liangjin Gong, Zeren Ma, Tao Xu

**Affiliations:** a Nanchang Hongdu Hospital of Traditional Chinese Medicine, Nanchang; b Gongqingcheng People's Hospital, Gongqingcheng, China.

**Keywords:** ankylosing spondylitis, protocol, systematic review, Yanghe decoction

## Abstract

**Background:**

Chinese herbal medicine is a commonly used traditional treatment for ankylosing spondylitis (AS). Among them, Yanghe decoction (YHD) has an obvious effect in relieving the symptoms of AS, but its efficacy is still controversial. The purpose of this study is to systematically evaluate the effectiveness and safety of YHD in the treatment of AS patients.

**Methods:**

From the establishment to September 2020, we will search a total of 7 electronic databases including PubMed, Cochrane Library, Embase, CNKI, VIP, WanFang, and the Chinese SinoMed Database. Two independent reviewers will search the database for relevant randomized controlled trials (RCTs), extract data, and evaluate the quality of the included RCTs. Data analysis will be processed by RevMan V.5.4 software.

**Results:**

The results of this systematic review will be published in a peer-reviewed journal.

**Conclusion:**

This study will provide evidence for the effectiveness of YHD in treating patients with AS.

## 1 Introduction

Ankylosing spondylitis (AS) is a common clinical chronic inflammatory immune disease. It is mainly manifested by low back pain and stiffness in the early stage. With the progressive development of the disease, spinal stiffness, deformity, and dysfunction may even occur, which will seriously affect the patient's quality of life.^[1,2]^ According to statistics, the prevalence of AS in China is about 0.3%, and it is more common among young people.^[3,4]^ AS is also one of the main reasons for the labor force loss of young and middle-aged people in China.^[5]^ Recent studies have shown that the occurrence of this disease is closely related to genetic, immune, environmental, infection, and endocrine factors.^[6,7]^ In addition, studies have shown that patients with AS may be associated with an increased risk of various diseases, such as type stroke, cardiovascular diseases, type 2 diabetes, chronic obstructive pulmonary disease, asthma, and depression.^[8]^

As far as the treatment of AS is concerned, the current main treatment goal of AS is to maintain the patient's physical function by controlling inflammation and symptoms, thereby preventing progressive structural damage and improving long-term quality of life.^[9]^ At present, most of the first-line drugs used to treat AS are non-steroidal anti-inflammatory drugs (NSAIDs), and they are recommended by clinical guidelines.^[10–12]^ In addition, glucocorticoids, anti-rheumatic drugs, and biological agents are also commonly used therapeutic drugs.^[7]^ Although the efficacy of drug therapy is definite, long-term drug therapy is usually accompanied by certain side effects, such as abdominal pain, upper gastrointestinal bleeding, loss of appetite, etc.^[8]^ Therefore, it is necessary to find a safe and effective alternative therapy to treat AS. Chinese herbal medicine (CHM), as a part of traditional Chinese medicine therapy, is a complementary and alternative therapy widely used in the world. Recent studies have shown that CHM can effectively relieve the symptoms of AS.^[3]^

Yanghe decoction (YHD) is a clinically widely used prescription, which consists of rehmannia, cinnamomum cassia, semen brassicae, deerhorn glue, ephedra, radix glycyrrhizae, and charcoal of ginger.^[13]^ Modern studies have shown that YHD has good anti-inflammatory and analgesic effects, and can effectively reduce muscle stiffness.^[3,14]^ Therefore, YHD is considered a safe and effective supplementary replacement therapy.

Although YHD is widely used in clinical practice, the effectiveness of YHD in the treatment of AS is still controversial. Moreover, there is still a lack of systematic reviews on the effectiveness of YHD on AS. Therefore, this study uses evidence-based medicine to analyze and evaluate clinical randomized controlled trials of YHD in the treatment of AS patients. This study will evaluate the effectiveness and safety of YHD in the treatment of AS.

## 2 Methods

This research has been registered on the Open Science Framework (OSF, https://osf.io/). The registered DOI of this study is 10.17605 /OSF.IO/74UQD. We will perform this research in accordance with Preferred Reporting Items for Systematic Reviews and Meta-analyses.

### 2.1 Inclusion criteria for study selection

#### 2.1.1 Types of studies

RCTs of YHD for the management of AS patients will be included. Non-RCTs and observational studies will be excluded.

#### 2.1.2 Types of participants

All AS patients in the included RCTs need to meet a clear diagnosis, and there are no restrictions on the race, sex, or age of AS patients.

#### 2.1.3 Types of interventions

##### 2.1.3.1 Experimental interventions

The intervention method of the treatment group is YHD or adjustment based on YHD. We have no restrictions on the treatment time and dosage of YHD.

##### 2.1.3.2 Control interventions

The treatment of the control group will include conventional symptomatic treatment with drugs, as well as no treatment and placebo. The control group including YHD will be excluded.

#### 2.1.4 Types of outcome measures

##### 2.1.4.1 Primary outcome

The primary outcomes will include clinical efficacy, symptom score, TCM syndrome score, and schober test outcome.

##### 2.1.4.2 Additional outcomes

The safety assessment will be considered an additional result.

### 2.2 Search methods for the identification of studies

We will search from the following digital databases including PubMed, Cochrane Library, Embase, CNKI, VIP, WanFang, and the Chinese SinoMed Database. The search time for all electronic databases ends in September 2020. The search terms used in the database include diseases (Ankylosing Spondylitis OR spondyloarthritis), intervention (Yanghe Tang OR Yanghe decoction), and research types (randomized controlled trial, random trials, controlled clinical trial). Table 1 shows the search strategy in the PubMed database.

**Table 1:** Search strategy used in PubMed database.

Number	Search items
1	Ankylosing spondylitis. ti, ab
2	spondyloarthritis. ti, ab
3	1 or 2
4	Yanghe decoction. ti, ab
5	Yanghe Tang. ti, ab
6	4 or 5
7	randomized controlled trial. ti, ab
8	random trials. ti, ab
9	controlled clinical trial. ti, ab
10	7 or 8–9
11	3 and 6 and 10

### 2.3 Data collection and analysis

#### 2.3.1 Selection of studies

**Figure 1:**
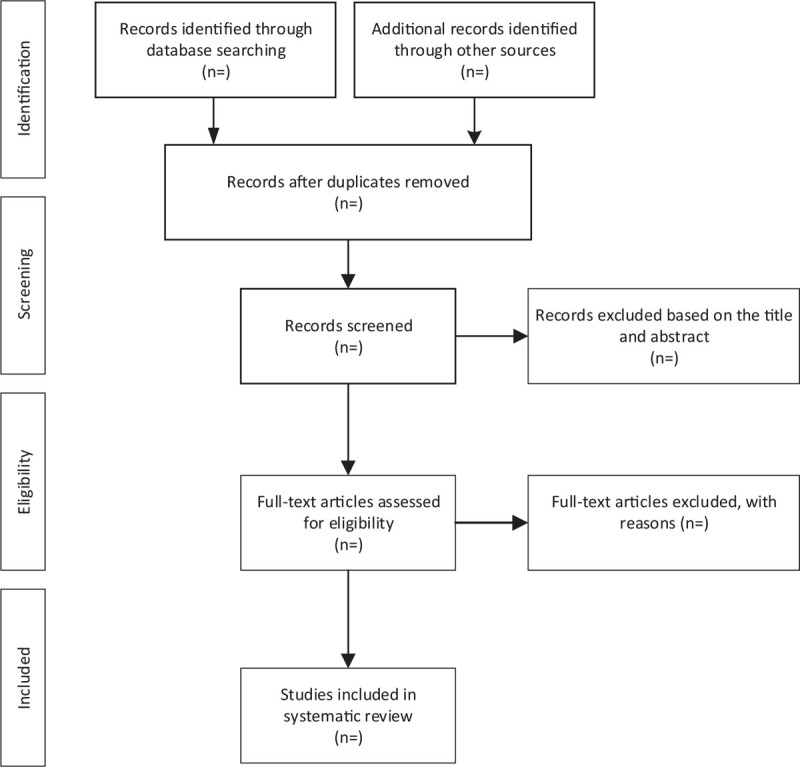
Flow diagram of study selection process.

We will conduct a literature search according to the search strategy, and import the citations retrieved from each electronic database into the EndNote X8 software for management, and then evaluate the eligibility of these articles according to the inclusion and exclusion criteria. The literature search will be done independently by 2 authors. If there is a disagreement, the 2 authors will discuss and negotiate with the third author. The selection process for the included studies is summarized in Fig. 1.

#### 2.3.2 Data extraction and management

Data from included articles will be extracted by 2 reviewers separately. Disagreements will be resolved through discussion with the corresponding author. The information we will extract will include the first author, publication year, participant characteristics, sample size, intervention measures, research results, and adverse events.

### 2.4 Risk of bias assessment

The Cochrane bias risk assessment tool will be used to assess the risk of bias (ROB) in the included studies. The assessment includes the following items: random sequence generation, allocation concealment, the blinding method for patients, researchers and outcomes assessors, incomplete result data, selective reports, and other sources of bias. The above 7 items will be assessed as high, unclear, or low risk of bias based on the information provided by the included studies.^[15]^ ROB will be evaluated by 2 independent researchers, and disagreements will be resolved through discussion.

### 2.5 Quantitative data synthesis and statistical methods

#### 2.5.1 Quantitative data synthesis

RevMan V.5.3.0 software (The Nordic Cochrane Center, The Cochrane Collaboration, 2014, Copenhagen, Denmark) will be used for data analysis. For continuous data, we will calculate with the mean difference (MD) of 95% CI, and for categorical data, we will calculate with the risk ratio (RR) of 95% CI.

#### 2.5.2 Assessment of heterogeneity

We will use chi-square test and *I*^*2*^ test to assess statistical heterogeneity. If the *P* value is >.10 and the *I*^*2*^ value is <50%, it indicates that the heterogeneity of the study is not obvious. Conversely, when the *P* value is <.10 or the *I*^*2*^ value is >50%, it indicates significant heterogeneity.

#### 2.5.3 Assessment of reporting biases

If >10 RCTs are included in this study, the funnel plots will be used to assess the potential publication biases. If <10 RCTs are included, the publication bias will be assessed by using the Egger test.

#### 2.5.4 Subgroup analysis

If the heterogeneity of the included RCTs is obvious, we will perform subgroup analysis through the different types of the control group.

#### 2.5.5 Sensitivity analysis

If we include sufficient RCTs, sensitivity analysis will be performed by excluding low-quality RCTs to assess the reliability and robustness of the meta-analysis.

#### 2.5.6 Grading the quality of evidence

The quality of the evidence will be assessed through the Grading of Recommendations Assessment, Development and Evaluation (GRADE), and the level of evidence will be classified as high, medium, low, or very low.^[16,17]^

## 3 Discussion

CHM is a commonly used alternative supplement therapy. Related research shows that CHM is beneficial to AS, and YHD is one of the representative prescriptions.^[3,18]^ Although previous RCTs have reported the efficacy of YHD in the treatment of AS, the effectiveness of YHD in the treatment of AS still lacks a comprehensive systematic evaluation. The purpose of this meta-analysis is to evaluate the efficacy and safety of YHD in the treatment of AS. The conclusions of this systematic review will provide evidence-based medicine recommendations for AS patients with YHD treatment.

## Author contributions

**Data curation:** Xiaosheng Xu, Hong Chen.

**Formal analysis:** Xiaosheng Xu, Yi Wan.

**Funding acquisition:** Xiaosheng Xu.

**Investigation:** Hong Chen, Xiaoping Yuan.

**Methodology:** Hong Chen, Liangjin Gong.

**Project administration:** Zeren Ma, Tao Xu.

**Resources:** Tao Xu.

**Software:** Xiaoping Yuan, Yi Wan.

**Supervision:** Tao Xu.

**Validation:** Liangjin Gong, Tao Xu.

**Visualization:** Liangjin Gong, Zeren Ma.

**Writing – original draft:** Xiaosheng Xu, Tao Xu.

**Writing – review & editing:** Xiaosheng Xu, Tao Xu.
